# Assessing the Readability and Comprehensibility of Online Patient Educational Materials for Common Psychotic Disorders

**DOI:** 10.7759/cureus.68064

**Published:** 2024-08-28

**Authors:** Joshua L Davis, Cameron Gerhold, Jason Roeder, Rachel A Carr, Lawrence Mobley

**Affiliations:** 1 Psychiatry, Florida State University College of Medicine, Tallahassee, USA; 2 Orthopedic Surgery, Florida State University College of Medicine, Tallahassee, USA; 3 Psychology, Florida International University, Miami, USA; 4 Pediatrics, The Johns Hopkins Hospital, Baltimore, USA

**Keywords:** psychotic disorders, mental health education, schizophrenia and other psychotic disorders, online patient education, flesch-kincaid, readability measures, psychiatry & mental health

## Abstract

Background

In our age of technology, millions of people use the Internet daily for health-related searches and guidance, both patients and caregivers alike. However, health literacy remains notably low among U.S. adults, and this issue is particularly critical for individuals with severe mental illnesses. Poor health literacy is often linked to low socioeconomic status and correlates with adverse patient outcomes and limited healthcare access. With the average reading level of the U.S. adult at the eighth-grade level, guidelines recommend health information be written to match. This study focuses on the readability of top Google search results for common psychotic disorders, emphasizing the need for accessible online health information to support vulnerable populations with severe mental illnesses.

Methods

The top five most visited websites for eight psychiatric conditions were included in this study. These conditions included schizophrenia, schizoaffective disorder, schizophreniform disorder, delusional disorder, bipolar 1 disorder, major depressive disorder (MDD) with psychotic features, substance-induced psychotic disorder, and psychotic disorder due to a general medical condition. The Flesch-Kincaid (FK) reading ease and grade level score were calculated for each webpage. Additionally, all institutions and organizations that created each webpage were noted.

Results

The average FK grade level was 9.9 (corresponding to a 10th-grade level), while the overall FK reading ease was 37.3 (corresponding to college-level difficulty) across all disorders analyzed. Websites on MDD with psychotic features had the lowest average FK grade level, 8.6, and best reading ease score. Websites discussing delusional disorder had the highest average FK grade level, 11.2, while those with information on schizophreniform disorder had the lowest average reading ease with a score of 31.7, corresponding to “difficult (college)” level reading.

Conclusion

Both patient education and compliance can be improved with more accessible and readable patient educational materials. Our study shows significant opportunities for improvement in the readability and comprehensibility of online educational materials for eight of the most common psychotic disorders. Physicians and other healthcare providers should be aware of this barrier, recommending specific websites, literature, and resources for patients and their caregivers. Further efforts should be aimed at creating new and easy-to-comprehend online material for mental health disorders, ensuring the best quality and care for these patients.

## Introduction

With the continued increase of available technologies and resources, we are now, more than ever, living in an age of technology. From smartphones to laptops, tablets, and artificial intelligence, the amount of available online information increases daily, often with minimal quality measures [1]. According to the National Center for Health Statistics, 58.5% of adults have used the Internet in the past 12 months to seek health or medical information [2]. While the Internet has the potential to serve as an informative learning tool, allowing patients and caregivers alike to better understand their health and various illnesses, not all online information is ideal.

Per the U.S. government’s Healthy People 2030 initiative, health literacy is defined as “the degree to which individuals have the ability to find, understand, and use information and services to inform health-related decisions and actions for themselves and others” [3]. It is estimated that only 12% of U.S. adults have proficient health literacy, with 43% of adults at the basic or below basic health literacy levels [4]. Inadequate health literacy is strongly associated with a low socioeconomic position [5] and has been shown to correlate with adverse patient outcomes [6].

According to a Gallup analysis collected using data from the U.S. Department of Education, roughly 130 million people, or 54%, of U.S. adults lack literacy proficiency, noting that more than half of the adult population ages 16 to 74 read at or below the sixth-grade level [7], with the average reading level of the U.S. adult at the eighth-grade level [8]. With this, organizations including the American Medical Association (AMA), Centers for Disease Control and Prevention (CDC), and National Institute of Health (NIH) have posted guidelines regarding health literacy and the readability of patient-related information. These guidelines recommend that health information and medical education material designed for the public be written no higher than the eighth-grade level, with the AMA even recommending written health information to be at or below the sixth-grade reading level [8,9]. Furthermore, individuals with low health literacy have shown to be significantly more likely to report difficulty finding accessible healthcare [10], as well as a negative correlation with a patient’s ability to evaluate online health information [11], highlighting the importance of readable and simple to understand health resources.

When searching for information online, particularly those that are medically related, patients often encounter content from a wide variety of sources - academic institutions, government organizations, medical journals, and private corporations. Despite the vast amount of online information available, a 2020 report based on over 80 million keyword searches within Google revealed that over 25% of click rates occur on the first Google search result [12]. Additionally, 70.4% of all clicks occurred within the top five searches [12], highlighting the significance of having reliable and readable information in the top five Google search rankings.

While often viewed as a subjective matter, there are objective ways to measure this readability, with the most commonly used metrics being the Flesch-Kincaid (FK) Reading Ease and Grade Level scores [13]. This scoring method uses sentence and word length to produce a numerical readability score, which can then be converted to determine the reading difficulty and estimated reading grade level needed to understand the given body of text [13]. The effectiveness of any resource depends on the audience's literacy and the material's readability, with FK scores providing a chance to align these factors.

This study aims to analyze the readability of the top five Google searches for eight of the most prevalent disorders associated with psychosis: schizophrenia, schizoaffective disorder, schizophreniform disorder, delusional disorder, bipolar 1 disorder, major depressive disorder (MDD) with psychotic features, substance-induced psychotic disorder, and psychotic disorder due to a general medical condition [14]. While the health and wellness of many are impacted by health literacy and socioeconomic factors, there is a significant and known negative correlation between socioeconomic status (SES) and mental illness [15]. With this, in combination with the known correlation between health literacy and SES [5], health outcomes [6], and healthcare accessibility [10], patients suffering from mental illnesses may be disproportionately affected by resources with readability scores beyond that of recommended guidelines. Our goal is to provide evidence supporting the need for readable online material for this vulnerable population, as our findings may highlight gaps in current online health information that need to be addressed to improve accessibility and understanding for a large group of individuals.

## Materials and methods

The Flesh-Kincaid (FK) grade level and FK reading ease, both highly validated tools used to assess the readability of texts, were calculated for the top five most visited websites on schizophrenia, schizoaffective disorder, schizophreniform disorder, delusional disorder, bipolar 1 disorder, MDD with psychotic features, substance-induced psychotic disorder, and psychotic disorder due to a general medical condition. In total, FK grade level and FK reading ease were calculated for 40 websites to assess their readability [16]. The Google search for all content was conducted by a single investigator on July 29, 2024. An incognito search was used, with all cookies, cache, and location cleared or turned off to optimize the generalizability of study results.

FK reading ease is a metric used to indicate how easy or challenging a text is to comprehend. Easier texts to understand have a higher FK reading ease, while texts that are more difficult to comprehend have a lower FK reading ease. It can be calculated using the following formula: 206.835 - 1.015 × (average number of words per sentence) - 84.6 × (average number of syllables per word) [16]. FK reading ease measurements can be interpreted at the following levels: 0-30 is very difficult (college graduate level), 31-50 is difficult (college level), 51-60 is fairly difficult (high school level), 61-70 is standard (eighth to ninth-grade level), 71-80 is fairly easy (seventh-grade level), 81-90 is easy (sixth-grade level), and 91-100 is very easy (fifth-grade level) [13].

The Flesh-Kincaid grade level is a measurement that determines the estimated completed grade level necessary for a reader to fully understand a body of text. FK grade level was calculated for all sites using the following formula: 0.39 × (average number of words per sentence) + 11.8 × (average number of syllables per word) - 15.59 [16]. All calculations are rounded up or down, depending on the number within the 10th place following the decimal. For example, an FK grade level calculation of 8.4 rounds down to eight (correlating to the text being written at an eighth-grade level). FK grade level scores ending in a decimal point of 0.5 or higher were rounded up, correlating to the increased grade level. The relationship between FK reading ease and the difficulty of a given text is inversely correlated, while the relationship between FK grade level and text difficulty is directly correlated.

## Results

**Figure 1:**
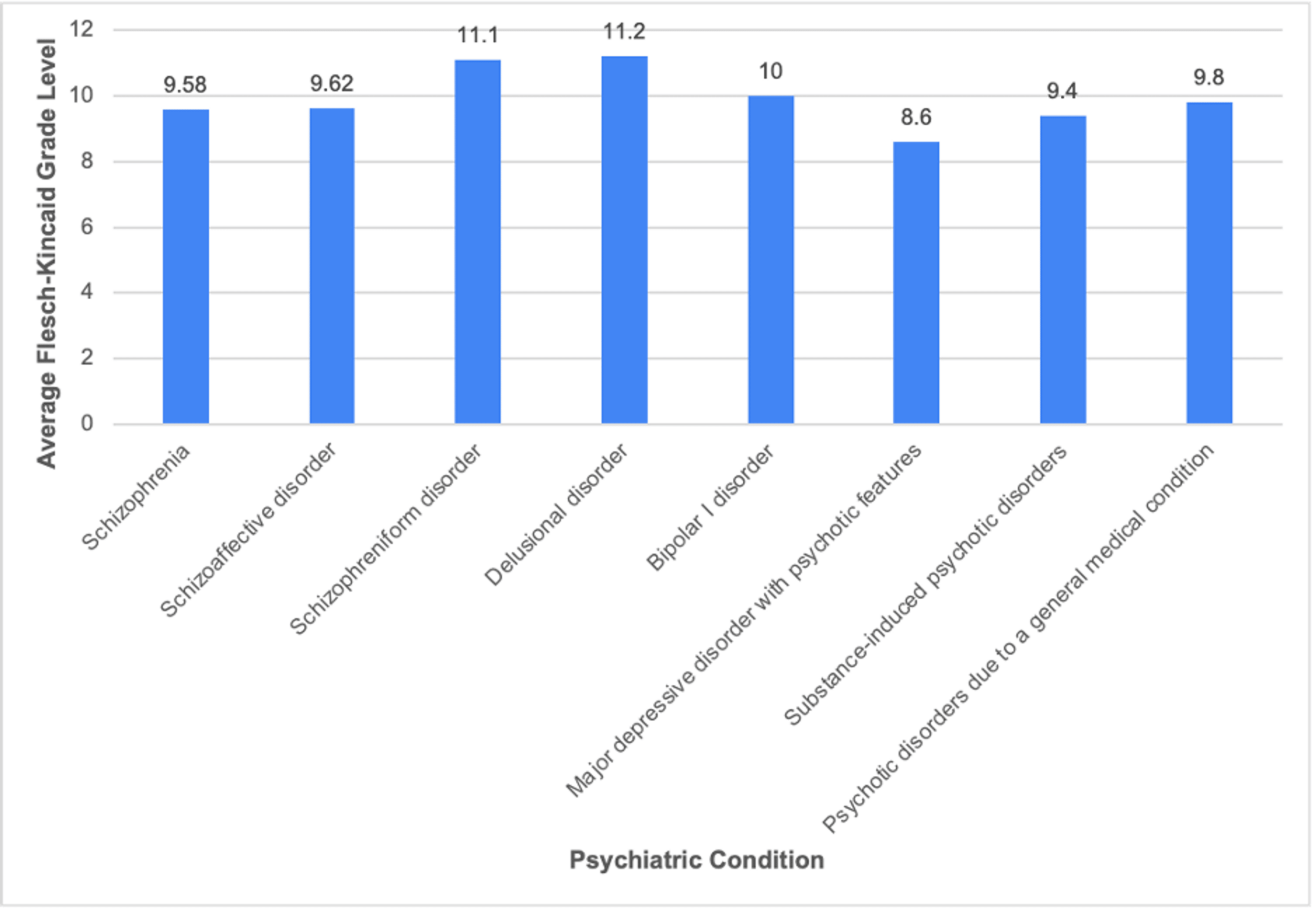
Average Flesch-Kincaid grade level of online patient educational materials for each psychiatric condition

**Figure 2:**
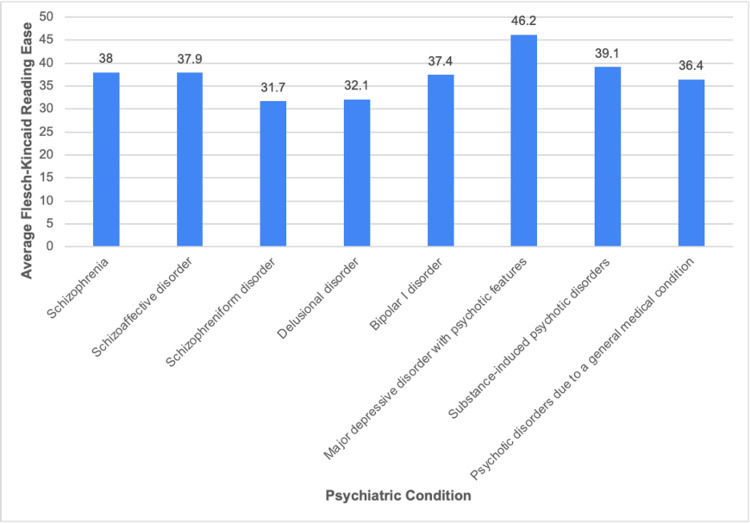
Average Flesch-Kincaid reading ease of online patient educational materials for each psychiatric condition

Figure 1 shows the average FK grade level scores of the five most visited websites for each of the eight psychiatric conditions assessed in this study. The overall average FK grade level for the 40 websites is 9.9. This score indicates that a 10th-grade reading level is required to effectively comprehend the content of these websites. Figure 2 shows the average FK reading ease scores for the five most visited websites for all eight psychiatric conditions. The overall average FK reading ease for all websites is 37.3, which corresponds with “difficult” or “college” level comprehensive ease [13].

Websites on MDD with psychotic features had the lowest average FK grade level, 8.6, and the best FK reading ease score, 46.2. This reading ease score did, however, remain at the college level of comprehension [13]. Websites on delusional disorder had the highest average FK grade level, 11.2. Websites providing information on schizophreniform disorder had the lowest average FK reading ease, with a score of 31.7, also equating to the college level.

Of the 40 web pages analyzed, all FK reading ease scores were above the sixth-grade reading level. The best reading ease score of any webpage analyzed was 54.3, corresponding to a high school level. Table 1 provides the FK reading ease, grade level, the corresponding organization or institution of publication, and website links for all pages analyzed in this study.

**Table 1:** Flesh-Kincaid reading ease scores, Flesch-Kincaid grade level scores, and websites analyzed for each psychiatric condition evaluated APA, American Psychiatric Association; FK, Flesh-Kincaid; MDD, major depressive disorder; NAMI, National Alliance on Mental Illness; NIMH, National Institute of Mental Health; NHS, National Health Service; NIH, National Institutes of Health; WHO, World Health Organization

Organization/Institution	Google Search Condition	FK Reading Ease	FK Grade Level	Website
NIMH	Schizophrenia	41.0	10.0	https://www.nimh.nih.gov/health/topics/schizophrenia [17]
Mayo Clinic	Schizophrenia	46.2	8.1	https://www.mayoclinim.org/diseases-conditions/schizophrensc/symptoms-causes/syc-20354443 [18]
APA	Schizophrenia	25.8	10.9	https://www.psychiatry.org/patients-families/schizophrenia/what-is-schizophrenia [19]
WebMD	Schizophrenia	38.2	9.6	https://www.webmd.com/schizophrenia/mental-health-schizophrenia [20]
WHO	Schizophrenia	38.8	9.3	https://www.who.int/news-room/fact-sheets/detail/schizophrenia [21]
Mayo Clinic	Schizoaffective disorder	43.0	8.4	https://www.mayoclinic.org/diseases-conditions/schizoaffective-disorder/symptoms-causes/syc-20354504 [22]
NAMI	Schizoaffective disorder	28.7	10.8	https://www.nami.org/about-mental-illness/mental-health-conditions/schizoaffective-disorder/ [23]
Cleveland Clinic	Schizoaffective disorder	39.8	9.8	https://my.clevelandclinic.org/health/diseases/21544-schizoaffective-disorder [24]
WebMD	Schizoaffective disorder	44.4	8.6	https://www.webmd.com/schizophrenia/mental-health-schizoaffective-disorder [25]
NIH	Schizoaffective disorder	33.6	10.5	https://www.ncbi.nlm.nih.gov/books/NBK541012/ [26]
WebMD	Schizophreniform disorder	39.3	9.0	https://www.webmd.com/schizophrenia/mental-health-schizophreniform-disorder [27]
Cleveland Clinic	Schizophreniform disorder	35.7	10.4	https://my.clevelandclinic.org/health/diseases/9571-schizophreniform-disorder [28]
Marck Manual	Schizophreniform disorder	13.0	16.5	https://www.merckmanuals.com/professional/psychiatric-disorders/schizophrenia-and-related-disorders/schizophreniform-disorder [29]
Sheppard Pratt	Schizophreniform disorder	34.8	9.6	https://www.sheppardpratt.org/knowledge-center/condition/schizophreniform-disorder/ [30]
Medical News Today	Schizophreniform disorder	35.9	10.0	https://www.medicalnewstoday.com/articles/schizophreniform [31]
Cleveland Clinic	Delusional disorder	29.4	11.7	https://my.clevelandclinic.org/health/diseases/9599-delusional-disorder [32]
WebMD	Delusional disorder	41.1	9.4	https://www.webmd.com/schizophrenia/delusional-disorder [33]
NIH	Delusional disorder	34.7	10.5	https://www.ncbi.nlm.nih.gov/books/NBK539855/ [34]
Merck Manual	Delusional disorder	31.4	13.2	https://www.merckmanuals.com/home/mental-health-disorders/schizophrenia-and-related-disorders/delusional-disorder [35]
Psychology Today	Delusional disorder	23.7	11.1	https://www.psychologytoday.com/us/conditions/delusional-disorder [36]
APA	Bipolar I disorder	23.9	11.0	https://www.psychiatry.org/patients-families/bipolar-disorders/what-are-bipolar-disorders [37]
WedMD	Bipolar I disorder	38.1	9.8	https://www.webmd.com/bipolar-disorder/bipolar-1-disorder [38]
NIMH	Bipolar I disorder	44.1	9.7	https://www.nimh.nih.gov/health/topics/bipolar-disorder [39]
Mayo Clinic	Bipolar I disorder	44.3	8.4	https://www.mayoclinic.org/diseases-conditions/bipolar-disorder/symptoms-causes/syc-20355955 [40]
Cleveland Clinic	Bipolar I disorder	36.7	11.0	https://my.clevelandclinic.org/health/diseases/9294-bipolar-disorder [41]
MedlinePlus	MDD with psychotic features	47.5	8.1	https://medlineplus.gov/ency/article/000933.htm [42]
NIH	MDD with psychotic features	43.9	9.8	https://www.ncbi.nlm.nih.gov/pmc/articles/PMC3572511/ [43]
Healthline	MDD with psychotic features	45.5	8.6	https://www.healthline.com/health/depression/psychotic-depression [44]
NHS	MDD with psychotic features	54.3	7.6	https://www.nhs.uk/mental-health/conditions/psychotic-depression/ [45]
WedMD	MDD with psychotic features	39.7	8.9	https://www.webmd.com/depression/psychotic-depression [46]
Sheppard Pratt	Substance-induced psychotic disorder	42.5	8.6	https://www.sheppardpratt.org/knowledge-center/condition/substance-induced-psychotic-disorder/ [47]
Merck Manual	Substance-induced psychotic disorder	22.0	11.4	https://www.merckmanuals.com/professional/psychiatric-disorders/schizophrenia-and-related-disorders/substance-medication-induced-psychotic-disorder [48]
American Addiction Centers	Substance-induced psychotic disorder	45.0	8.6	https://americanaddictioncenters.org/co-occurring-disorders/drug-psychosis-comorbidity [49]
Greenhouse Treatment Center	Substance-induced psychotic disorder	42.6	9.1	https://greenhousetreatment.com/co-occurring-disorders/drug-induced-psychosis/ [50]
NIH	Substance-induced psychotic disorder	43.2	9.2	https://www.ncbi.nlm.nih.gov/pmc/articles/PMC8732862/ [51]
Psychology Today	Psychotic disorders due to a general medical condition	23.6	11.1	https://www.psychologytoday.com/us/conditions/psychotic-disorder-due-another-medical-condition [52]
Merck Manual	Psychotic disorders due to a general medical condition	22.6	11.7	https://www.merckmanuals.com/professional/psychiatric-disorders/schizophrenia-and-related-disorders/psychotic-disorder-due-to-another-medical-condition [53]
WebMD	Psychotic disorders due to a general medical condition	45.0	8.6	https://www.webmd.com/schizophrenia/mental-health-psychotic-disorders [54]
Cleveland Clinic	Psychotic disorders due to a general medical condition	45.3	9.3	https://my.clevelandclinic.org/health/symptoms/23012-psychosis [55]
NHS	Psychotic disorders due to a general medical condition	45.3	8.3	https://www.nhs.uk/mental-health/conditions/psychosis/causes/ [56]

Schizophrenia

The average FK grade level of the webpages for schizophrenia was 9.6, with a standard deviation of 1.0. All websites on schizophrenia were determined to be above the sixth-grade reading level, the recommended reading level set forth by the AMA [8]. Only one website was written at an eighth-grade reading level, which is the maximum recommended reading difficulty level for healthcare information per the CDC and NIH [9]. The average FK reading ease score for the five websites analyzed was 38.0. This correlates to difficult/college-level comprehension.

Schizoaffective disorder

The average FK grade level of the webpages for schizoaffective disorder was 10.5, with a standard deviation of 1.1. All websites on schizoaffective disorder were determined to be written at a reading level above sixth grade. Only one website was written at an eighth-grade reading level. The average FK reading ease score for the five websites analyzed was 33.6. This correlates to difficult/college-level comprehension.

Schizophreniform disorder

The average FK grade level of the webpages for schizophreniform disorder was 11.1, with a standard deviation of 3.1. All websites on schizophreniform disorder were determined to be written at a reading level above a sixth-grade reading level. Additionally, no websites were written at an eighth-grade reading level. The average FK reading ease score for the five websites analyzed was 31.7. This correlates to difficult/college-level comprehension.

Delusional disorder

The average FK grade level of the webpages for delusional disorder was 11.1, with a standard deviation of 1.4. All websites on delusional disorder were determined to be written at a reading level above a sixth-grade reading level. Furthermore, all websites were written above an eighth-grade reading level. The average FK reading ease score for the five websites analyzed was 32.1. This correlates to difficult/college-level comprehension.

Bipolar 1 disorder

The average FK grade level of the webpages for bipolar 1 disorder was 10.0, with a standard deviation of 1.1. All websites on bipolar 1 disorder were determined to be written at a reading level above a sixth-grade reading level. Only one website was written at an eighth-grade reading level. The average FK reading ease score for the five websites analyzed was 37.4. This correlates to difficult/college-level comprehension.

MDD with psychotic features

The average FK grade level of the webpages for MDD with psychotic features was 8.6, with a standard deviation of 0.8. All websites on MDD with psychotic features were determined to be written at a reading level above a sixth-grade reading level. However, two websites were written at an eighth-grade reading level. The average FK reading ease score for the five websites analyzed was 46.2. This correlates to difficult/college-level comprehension.

Substance-induced psychotic disorder

The average FK grade level of the webpages for substance-induced psychotic disorder was 9.4, with a standard deviation of 1.2. All websites on this condition were determined to be written at a reading level above a sixth-grade reading level. Additionally, all websites were written above an eighth-grade reading level. The average FK reading ease score for the five websites analyzed was 39.1. This correlates to difficult/college-level comprehension.

Psychotic disorder due to a general medical condition

The average FK grade level of the webpages for psychotic disorder due to a general medical condition was 9.8, with a standard deviation of 1.5. All websites on this condition were determined to be written at a reading level above a sixth-grade reading level. Only one website was written at an eighth-grade reading level. The average FK reading ease score for the five websites analyzed was 45.3. This correlates to difficult/college-level comprehension.

## Discussion

As a whole, schizophreniform and delusional disorder had the highest FK grade-level scores, both scoring at the 11th-grade level with reading ease scores of 31.7 and 32.1, respectively. This was followed by bipolar 1 disorder, schizophrenia disorder, schizoaffective disorder, and psychotic disorders due to a general medical condition, all of which averaged a 10th-grade reading level with reading ease scores rated as “difficult.” Both substance-induced psychotic disorder and MDD with psychotic features averaged ninth-grade reading levels; however, reading ease remained in the “difficult” range for comprehension [13].

Overall, the average reading levels across all disorders analyzed exceed the sixth- to eighth-grade reading levels suggested by the AMA, CDC, and NIH for creating easy-to-read written materials [8,9]. Of the 40 web pages analyzed, six sites were scored at or below the eighth-grade level. Of note, two web pages analyzed in this study were published by the National Health Service (NHS) of England. Both NHS sites scored well regarding their readability, with one at the seventh-grade level and one at the eighth-grade level. All disorders analyzed averaged FK reading ease scores that are considered “difficult” and “college level” reading [13].

These results highlight a significant downfall of web-based health-related information for psychotic disorders, as the majority of sites accessed by the public may be difficult to read and comprehend. Interestingly, these results may be even more significant for patients with severe mental illness, as cognitive dysfunction is often a key feature in many psychiatric disorders [57]. This dysfunction is even greater in patients with psychotic disorders, particularly schizophrenia, and similar illnesses [58]. A 2014 study published in the American Journal of Psychiatry indicates that patients with schizophrenia show severe deficits in reading abilities compared to matched controls [58]. This study also notes that over 70% of patients from the schizophrenia group met the criteria for dyslexia, with 50% of patients reading below the eighth-grade level despite intact premorbid reading ability [58], highlighting the commonality and possibility of worsening cognitive declines in patients with psychotic disorders. Furthermore, there is continued evidence suggesting baseline lower health literacy levels in patients with severe mental illness [59].

As the accessibility of online and personalized health information continues to grow, it is essential that information be tailored to diverse patient populations. In psychotic disorders, there is often a significant delay between the onset of symptoms and the initiation of treatment. This delay is often greater than one year [60,61] and can be correlated to worsened treatment response and outcomes [62]. Many factors, including stigma, the complexity of symptoms, and the accessibility and comprehension of the available information, may influence such delays. Inadequate understanding of disease symptoms and treatment options can lead to further delays in necessary treatments, continuing to highlight the need for creating easily understandable online health resources that can bridge the gap between untreated symptoms and effective intervention in this vulnerable population.

Additionally, with compliance and relapse rates being a significant issue in psychotic disorders and their treatments [63], having readable, easy-to-comprehend educational material available for these patients and their caregivers is essential. There is a known increased risk of premature mortality in patients with severe mental illness [64]. However, the cause of this is multifactorial, including individual, community, and socioeconomic factors. Increased patient education has proven to significantly improve compliance levels across many chronic diseases and illnesses [65], and this should be further examined regarding severe mental health conditions. Organizations and writers of educational material for mental health disorders must be aware of the language and format they use to provide content. Information needs to be written in a manner that is easily read and understood by the majority, as the negative implications of poor patient education reach beyond that of initial realization. When educational materials exceed the recommended guidelines set forth by the AMA, CDC, and NIH, we may unintentionally propagate the disparities in health literacy and health education for patients with severe mental health disorders and their caregivers, ultimately hindering their treatment outcomes and overall well-being.

Limitations

Patients may experience considerable variability in websites when seeking health information. This variability can depend on factors such as search engine, geographic location, device used to search, language, and search history. For this study, we used the Google search engine. While Google is the most popular search engine, patients may use others, such as Yahoo, Bing, and Ask.com, leading to different results. Therefore, we could not account for variability in search results across various search engines or based on individual search algorithms. Furthermore, this study analyzed only the top five search results, which may not provide a comprehensive overview of all available information. Additionally, the readability scoring used in this study, specifically the FK readability scores, does not account for the layout of text/websites, pictures, or charts that may impact comprehension. These measures also do not account for prior knowledge, motivation, and access to other resources, which also affect a person's ability to read and comprehend text.

## Conclusions

In conclusion, the readability and comprehensibility of online educational materials for eight of the most common psychotic disorders remain beyond the recommended guidelines set forth by the AMA, CDC, and NIH. Additionally, cognitive dysfunction and baseline decreased health literacy are often noted in many severe mental health disorders, underscoring a potentially increased effect on these patients. Patient education plays a significant role in treatment compliance, highlighting a potential opportunity to create a positive change in the course of these disorders. Physicians should be aware of specific webpages with trustworthy and comprehensible health education material and recommend these resources to patients and caregivers. Further projects and studies should be aimed at creating new and easy-to-comprehend online material for mental health disorders.
